# In Situ Monitoring of Additive Manufacturing Using Digital Image Correlation: A Review

**DOI:** 10.3390/ma14061511

**Published:** 2021-03-19

**Authors:** Filipa G. Cunha, Telmo G. Santos, José Xavier

**Affiliations:** UNIDEMI, Department of Mechanical and Industrial Engineering, NOVA School of Science and Technology, NOVA University Lisbon, 2829-516 Caparica, Portugal; fa.cunha@campus.fct.unl.pt (F.G.C.); telmo.santos@fct.unl.pt (T.G.S.)

**Keywords:** digital image correlation, in situ, monitoring, additive manufacturing

## Abstract

This paper is a critical review of in situ full-field measurements provided by digital image correlation (DIC) for inspecting and enhancing additive manufacturing (AM) processes. The principle of DIC is firstly recalled and its applicability during different AM processes systematically addressed. Relevant customisations of DIC in AM processes are highlighted regarding optical system, lighting and speckled pattern procedures. A perspective is given in view of the impact of in situ monitoring regarding AM processes based on target subjects concerning defect characterisation, evaluation of residual stresses, geometric distortions, strain measurements, numerical modelling validation and material characterisation. Finally, a case study on in situ measurements with DIC for wire and arc additive manufacturing (WAAM) is presented emphasizing opportunities, challenges and solutions.

## 1. Introduction

Additive manufacturing (AM), also known as 3D printing, is the process of creating a part by joining material, typically by adding news layers over a substrate, in order to obtain a final product from data in a computer-aided design (CAD) model [1]. The AM technology has gained enormous industrial and research interest in recent years, due to its potential for manufacturing complex single-stage components [2]. In the last decade, this technology has come to be seen as a process for manufacturing functional parts for a wide range of polymers and metallic materials. However, as AM typically has a low production rate, it is more suitable for manufacturing specific and complex parts than mass production. It is a technique successfully applied across different areas, from biomedical [3] to electric motors [4] to aerospace industries [5]. Another technological advantage is the ability to produce parts with an almost final shape, thus reducing production time and costs [6]. Nevertheless, products obtained by AM still require further development on open issues regarding internal defects due to printing errors and residual and thermal stresses, for instance [7,8]. This quality uncertainty can create parts wastage, increase parts delivery time and prevent AM implementation in industries that require high performance components with quality assurance.

In the paradigm of Industry 4.0, a tight integration of AM processes has been established regarding new classes of advanced materials. Examples of advanced materials are, for instance, Functionally Graded Materials (FGM) and hybrid material parts that can have an outstanding mechanical behaviour regarding homogeneous standard engineering counterparts. The hypothesis may be formulated from a biological perspective. Consistent progress of AM technology will yield the synthesis of materials with specific-oriented structure and functionality. This approach claims an unprecedented potential in which materials can evolve into the bio-inspired paradigm of heterogeneity and gradient mechanical properties [9]. The design of advanced materials exhibiting exceptional characteristics can now be properly tackled by AM processes towards application-driven engineering solutions. This vision shares the same paradigm as the patient-specific healthcare systems and medical devices. In addition, this new generation of high-value materials will contribute to environmental and sustainability challenges by the rational use of resources and energy.

In the onset of the so-called digital revolution, image-based technologies have been emerging. This progress has allowed the development of full-field optical techniques (FFOTs) for experimental continuum mechanics [10,11]. Intrinsically, these methods are in contrast with counterpart devices by providing contactless and full-field data. According to the physical phenomenon involved in the image formation, these methods can be divided into two main categories: white-light (e.g., digital image correlation, grid method, projection moiré) and interferometric (e.g., moiré interferometry, electronic speckle pattern interferometry, speckle shearography) techniques [12,13]. On the one hand, white-light techniques rely on the analysis of the light intensity variation of a geometrical pattern describing the material deformation. These types of techniques can be sorted regarding the characteristic pattern as random, period or feature-based. On the other hand, the interferometric techniques are based on the phenomenon of interference of light waves. These techniques use a monochromatic and coherent light source (e.g., a laser) to illuminate the material surface. Considering the way light interacts with this surface, these methods can be sorted into diffused light (speckle) and diffracted light (grating) interferometric techniques. The selection of a given optical technique is dependent on the application itself. Nevertheless, some guidelines may be defined considering the cost, the simplicity of implementation, the performances (spatial and temporal resolutions, accuracy), the kinematic quantity to be measured (e.g., displacement, strain, curvature), the expected range of deformation and the sensitivity to vibrations and the size of the region of interest (from nano to structural scales). The impact of FFOTs in providing kinematic measurements across a whole region of interest has brought novel insights into different applied scientific areas. Among others, one may underline the measurement of gradient fields [14,15,16], fracture cracking evaluation [17,18,19,20,21], image-based approaches of high-strain rate dynamical behaviour of materials [22,23,24,25,26], numerical modelling validation from image-based measurements [27,28,29] and inverse material parameter identification from heterogeneous tests [30,31,32,33]. Advanced monitoring techniques have also been applied with the AM process in order to enhance the technology.

This review aims at highlighting relevant achievements in applying image-based technologies for in situ monitoring during AM processes, with the scope of enhancing both the manufacturing process and the produced parts. The overview will be narrowed down to the DIC technique because of its current relevance as a full-field technique in this framework. Moreover, measurements regarded as in situ will be classed with regard to the scope of the study, highlighting the research interest and relevance for the AM processes. The DIC principle was recalled and its applicability to cope with in situ observations and measurements in different AM variants analysed. A critical discussion in applying DIC to AM processes in then presented on the light of key features concerning defect characterisation, residual stresses, geometric distortions, strain measurements, numerical validation and material characterisation. Finally, as an experimental case study, the feasibility to carry out in situ observations during the wire and arc additive manufacturing (WAAM) process was presented, highlighting challenges, recommendations and opportunities. The AM variants were summarised and briefly described in Appendix A.

## 2. In Situ Monitoring Using Full-Field Measurement Techniques

**Figure 1:**
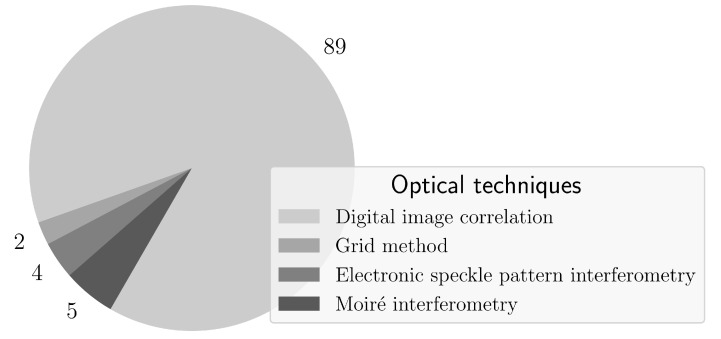
Number of publications in Scopus for different full-field optical techniques coupled with the searching keyword in situ measurements (units: %).

**Figure 2:**
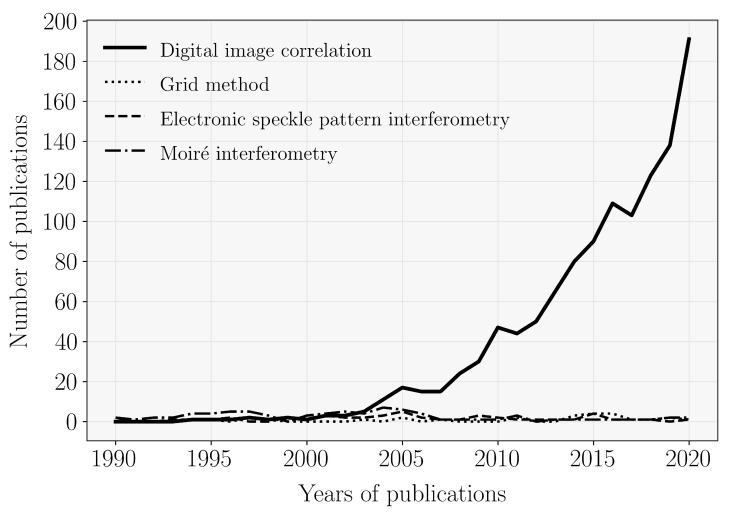
Number of publications for the different optical techniques with in situ measurements.

Several FFOTs are currently available providing full-field deformation measurements across a whole region of interest, for different purposes. They can be applied over a spectrum of different magnifications, from micro to structural scales of observation. For the purpose of this review, by in situ, it is understood monitoring that are taking place during a physical event. Hence, a research question can be raised concerning what optical techniques will be most suitable or have been already applied for in situ monitoring and for what specific purpose. A survey of published research on the topic and oriented review was carried out to answer this question. The SciVerse Scopus database was used to collect relevant publications using predefined searching keywords referring both to a given optical technique and in situ measurement. This strategy was adopted for the sake of reproducibility in defining the scientific methodology. A search for title, abstract and keywords was performed assuming DIC, grid method, electronic speckle pattern interferometry (ESPI) and moiré interferometry. Other optical techniques were not found relevant for the analysis. Figure 1 shows, in percentage, the amount of publications fulfilling such criteria. Generically, it can be concluded that DIC is the most used optical technique for in situ measurements. Furthermore, Figure 2 shows the evolution over time, since the year of 1990, of the number of publications crossing the searching keywords of the specific FFOT and in situ measurement. As it can be reinforced, there has been an increasing interest on DIC, particular in recent years, when compared to other counterpart FFOTs. Therefore, in the following, only the DIC technique will be actually addressed.

**Figure 3:**
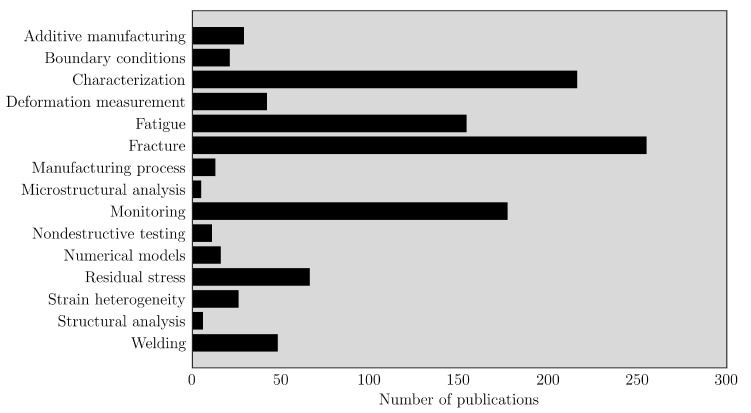
Applications of in situ measurements using DIC.

The term in situ measurements is still rather general and therefore needs to be further specified. A database survey was carried out to understand the purpose of studies coping with methodology based on in situ DIC measurements. Figure 3 summarised this analysis. In this case, several research topics were identified and articles classed according to the following topics, understood as the most comprehensive: AM, boundary conditions, characterisation, deformation measurements, fatigue, fracture, manufacturing process, microstructural analysis, monitoring, nondestructive testing, numerical models, residual stress, strain heterogeneity, structural analysis and welding.

In studies tackling in situ measurements in AM, two distinct objectives can be outlined. On the one hand, the scope was to perform in situ DIC measurements during the AM process itself, in order to obtain the part deformation when adding new layers of materials. This information can be relevant, for instance, to optimise the manufacturing parameters of the process in view of mitigating residual stresses and geometrical distortions, and to validate numerical simulation of the process and manufactured parts. On the other hand, the goal can be focused on the material characterisation of the manufactured parts by AM processes.

In the remaining topics, some studies enhanced classical data reduction methodologies, by integrating in situ data in the form of real boundary conditions. This information was relevant to enhance finite element analysis by considering both real geometry and boundary conditions, in contrast with prescribing idealised displacements at the boundary domain [34,35]. Full-field data were conveniently used in other studies for advanced inverse material characterisation, particularly dealing with heterogeneous stress and strain states that can be generated by geometric discontinuities, loading configurations or material structure [16,32,36]. In situ DIC measurements have also been playing an important role in fracture and fatigue problems. In particular, the kinematic field around the crack tip has been quantified and relevant mechanical parameters extracted from local full-field measurements including crack path and length, stress intensity factors, J-integral and cohesive laws [18,37,38,39,40,41,42]. In situ measurements were also conveniently carried out at lower scales of observation. At the microstructural level, heterogeneous strain can be revealed governing the mechanical behaviour of the material under tensile loading [5,43]. The image-based approach was most relevant at lower length scale, where conventional punctual techniques are not applicable. In situ monitoring were also used as a non-destructive testing (NDT) to inspect product manufacturing, installation and post-maintenance [44]. Finally, there are other studies in which in situ measurements by DIC were applied in the welding process to investigate residual stress formation, in contrast with the classical hole-drilling residual stress measurement technique [45,46].

### Digital Image Correlation Based Techniques

Several references in the literature can be pointed concerned with the DIC technique and principle [47]. Generically, three different optical configurations can be proposed known as 2D DIC, 3D DIC (stereovision) and digital volume correlation (DVC). A survey on the 2D DIC version can be found in [48], which covers the main principle and methodology to obtain displacement and strain fields from image correlation. Detailed discussions are given concerning measurement accuracy from both experimental conditions and algorithm details. Measures to achieve high accuracy deformation measurements are also recommended. This review was recently updated covering historical achievements, recent advances and open questions for further developments [49]. These articles are rather theoretical and therefore no practical code implementation is given. A recent paper in [50] aimed to overcome this gap, discussing code implementation in a scripting language for 2D DIC. An overview of the principle and applications of the stereovision set-up for experimental mechanics, which employees two cameras to measure 3D displacement and strain fields on any 3D object, is presented in [51]. Finally, a review of DVD can be found in [52], in which achievements, challenges, sources of measurement bias and uncertainties were analysed and ex-situ and in situ experiments discussed.

The 2D/3D DIC technique provides full-field, contactless displacements of a target object by correlating images recorded before and after the application of a given deformation. The subset-based correlation method is typically implemented in most available codes, although other approaches have been proposed such as the (global) finite element-based DIC [53] and the Fourier-based DIC [54]. There are several commercial software available in the market including Correlated Solutions [55], GOM Correlate [56], MatchID [57], LaVision [58] and Eikosim [59]. Open-source codes have also been developed such as Ncorr [60], DICe [61], DIC [62], multiDIC [63], pyxel [64], py2DIC [65], UFreckles [66] and YADICS [67]. In the subset-based approach, the reference (or undeformed) image is typically divided into subsets with may overlap by a step size. These are basic parameters that will influence the spatial resolution and accuracy of the measurements. Therefore, their selection typically requires a convergence study, in a compromise between correlation (small subsets) and interpolation (large subsets) errors [16,68,69]. Several mathematical correlation criteria have been proposed for estimating the displacement field in the subset matching algorithm approach [48]. It has been shown that the zero-normalized sum of squared differences (ZNSSD) is a robust algorithm since it takes into account both offset and linear scale variations of light intensity. The correlation criterion is solved with regard to deformation parameter which will defined the mapping function. An iterative algorithm, such as Newton–Raphson or Levenberg–Marquardt, can then be used for finding the optimal set of deformation parameters for the correlation coefficient.

DIC provides displacements at a large set of discrete data points across a surface of interest. However, strain fields are usually required in the mechanical analysis. Therefore, a robust (with regard to noise) numerical differentiation algorithm is needed to reconstruct the strain fields from measured displacements. Typically, this is achieved by means of function approximation, which can be locally or globally defined. In the local approach, a point-wise least square fitting strategy can be implemented [48,70]. The regularisation parameters in this case is the size of the strain window, which need to be selected by compromise between filtering and representativeness.

## 3. In Situ Monitoring of AM Using DIC

### 3.1. Applications

Among the scientific publications addressing in situ measurements using DIC in AM, a systematic review is presented highlighting objectives, methodologies, results and conclusions. The discussion will be outlined according the following research topics:

- Defect characterisation;
- Evaluation of residual stresses;
- Geometric distortions;
- Numerical modelling validation based on in situ measurements;
- Monitoring and part characterisation.

#### 3.1.1. Defect Characterisation

In the direct metal laser sintering (DMLS), Bartlett et al. [2] used a monitoring technique to quantify the relationships between the irregular geometry of the powder bed and the probability of defect formation. This approach provided a positive correlation aiming evaluation, characterisation and detection in the process. Based on 3D DIC measurements, speckled images were correlated using the Vic-3D software to extract the topology of the powder bed surface. The geometric topology and anomaly detection data were subsequently analysed. This work provided a new quantitative understanding of the importance of the quality of the powder bed in the formation of defects in the manufacture of metal additives. It was concluded that the problems of the powder layer were strongly related to the formation of physical defects in all components. In addition, it was determined that the size of these powder layer anomalies was directly correlated with the propensity to form defects. It was difficult, however, to predict the possible size of the physical microstructural defects that will be formed from the dimensions of the powder error at this junction. Nevertheless, using 3D DIC monitoring together with the Naïve–Bayes classification supervised by machine learning, it was possible to provide a prediction of defect formation, and therefore to take corrective actions during the production of the part. This type of information can be used in future work to correct critical powder problems during production. Currently, there are selective laser melting (SLM) machines on the market with corrective in situ monitoring that can identify the complete lack of powder cover on a part by sending a command to return to spread the powder layer. However, these feedback control systems cannot correct powder problems that do not fully expose the underlying part and have no understanding of the effect that the anomaly will have on defect formation and part properties. This work applies specifically to the propensity of defect formation in the presence of a powder bed irregularity and, of course, cannot predict defect formation in relation to other formation mechanisms.

Holzmond et al. [71] employed 3D DIC system as a non-destructive in situ measurement technique to monitor the surface of a fused filament fabrication (FFF) AM geometry. This study demonstrates the detection of local and global defects. The parts were manufactured using the FFF/fused deposition modelling (FDM) AM technique, also known as material extrusion. The authors used a stationary reference of known size and position to be created with each layer, and then the references can be correlated with each other and with the model file. This suggestion would eliminate the assumption that the bottom layer is flat and the need to rely on a low-quality patch pattern when determining the correlation between the part and the model data. As a result, there would be a decrease in calculation time and the need for optimal lighting.

#### 3.1.2. Evaluation of Residual Stresses

Due to the severe thermal gradients associated with some AM process, large residual stresses can be generated that will induce geometrical distortions in the manufactured parts, compromising its functionality. The integration of in situ measurements, provided by 3D DIC, to tackle this issue has been proposed by Bartlett et al. [72] for the SLM process. Despite the research interest in this field, it remains a challenge to measure the distribution of residual stresses from in situ monitoring techniques. Nevertheless, DIC has proven to be effective when compared to other NDT techniques when dealing with evaluation of residual stresses. To calculate residual stresses, it is necessary to obtain 3D surface measurements during or after the AM process. Thus, the authors proposed an inverted cone geometry for the sample. The results showed that the heterogeneous distribution of residual stresses was manifested by the reheating and sequential cooling of the new layer and changed dynamically between the layers. Finite element modelling validated the heterogeneous development of residual stresses. The stress state became more heterogeneous as more layers were added. Experimentally, it was concluded that the residual stresses has a strong dependence on the geometry of the part. It was concluded that the DIC measurement methodology developed for this experimental test has the necessary precision to evaluate the development and distribution in the AM parts.

#### 3.1.3. Geometric Distortions

Biegler et al. [73] presented a new approach based on full-field measurements to experimentally quantify the distortions of parts obtained by laser metal deposition (LMD) AM. Moreover, full-field data were used to validate the numerical simulation of the AM process [74]. In this case, the in situ DIC measurements were advantageously applied to measure distortions on the wall geometry produced by LMD. A comparison between the experimental and numerical results showed a good agreement between the direction of the sample length and the quantitative deviations in the direction of the height, which were attributed to the material model implemented in numerical the analyses. This study evidenced the adequacy of the in situ experimental approach to both inspect the geometrical quality of parts manufactured by LMD AM and to validate the numerical simulations. The model presented in this study can be eventually used to prevent distortions in the geometry of LMD parts if a complete calibration of the model and a critical discussion about the influence of the material properties are conducted. This was considered the first publication with internal distortion measurements directly on the geometry of an LMD AM metal during its construction.

Biegler et al. [75] validate a finite element model of a curved thin-walled manufactured by LMD by comparing the geometrical distortions obtained by means of in situ DIC measurements. The method generates reproducible results for transient distortions along all *x*, *y* and *z* directions. The most pronounced geometrical deviation was the *y* distortion, due to the deposition of layers along this direction. These data were then used to validate a thermomechanical simulation model. After calibrating the heat input, displacements were calculated for the entire construction and showed a good agreement both qualitatively and quantitatively with regard to the experimental data. The calibrated finite element model can then be employed as a predictive tool across regions of the manufactured parts where no experimental measurements were available. This is the case, for instance, when new layers are added, and no speckled pattern is presented for DIC measurements, or along the other face of the part without monitorisation. However, in order to extend the model to different parts, the calibration of the heat input must be redone to new parameters and the thickness and width must be adjusted to match the experiment.

Xie et al. [76] successfully obtained full-field deformation measurements during the laser engineered net shaping (LENS) AM process using DIC. The evolution of the vertical and longitudinal deformations of the material during the deposition process was accessed. An MLS2000 semiconductor laser was used for AM. Argon shielding gas was applied to protect the surface from oxidation during the manufacturing process. The results show that the longitudinal deformation increases rapidly with the tensile strain as the laser beam approaches, while the vertical deformation decreases rapidly.

#### 3.1.4. Numerical Modelling Validation Based on In Situ Measurements

Full-field measurements have been conveniently proposed to validate numerical models for general purposes [77]. This approach has also been followed using in situ measurements to validate numerical simulations on the AM process. These numerical models can be relevant to predict the mechanical behaviour of parts during the manufactured process itself. Biegler et al. [74] validated a structural thermomechanical simulation model in a direct energy deposition (DED) part using in situ measurements. The validation of an elasto-plastic finite element model for the deposition of directed energy from AM was shown in an arbitrarily curved geometry. The transient elasto-plastic finite element simulation was conducted in the commercial software Simufact.welding 7.1. The numerical simulation model was calibrated against the experimental data. The simulation results were compared with the in situ distortion measurements with good agreement.

Xie et al. [78] studied the evolution of transient deformation and thin wall distortion during AM using both in situ DIC measurements and numerical simulation. The results indicated that the evolution of the deformations varied with the location of the thin wall, especially in the centre and at the lateral ends. The non-uniform distribution of longitudinal and vertical deformations caused distortion of the thin wall during the manufacturing process. The distortion of the thin wall was predicted by the numerical method, which exhibits an inward contraction at the lateral ends and a downward contraction at the centre of the upper area. In this study, the Abaqus software was used to perform the finite element analysis of the procedure. This study consolidates the basis in using DIC and numerical simulation to predict and control distortions of parts during the AM process.

#### 3.1.5. Monitoring and Part Characterisation

He et al. [79] presented a work on in situ monitoring and characterisation during the direct metal deposition (DMD) processes with powder feed. For the AM industry, process stability and product quality are critical and are the main obstacles for manufacturers to adopt AM technologies, especially for high value and high precision applications. To improve the quality of AM processes, the monitoring and characterisation approaches with in situ processes are important for real time process control. Hence, it is possible to build a model with the parameters of the processes, allowing for estimating the control of the process as well as its optimization, predicting the characteristics of a final piece.

### 3.2. Customisation of the DIC to AM Process: Challenges and Current Solutions

Based on the state-of-the-art regarding the integration of in situ DIC measurements on AM processes, it is remarked that there are inherently several challenges in the practical implementation of the technique. This section outlines these difficulties and possible overcoming solutions. Table 1 summarises this analysis.

#### 3.2.1. Optical and Lighting System

The basic optical system for 2D DIC consists of a digital camera coupled with a suitable lens and a dedicated illumination. There are two types of digital sensors used in the camera technology: CCD (Charge-Coupled Device) and CMOS (Complementary Metal Oxide Semiconductor) [80]. CCD sensors have a limit on reading speed because they read data from each pixel through a single reading, whilst CMOS sensors can read data from several pixels simultaneously. The power consumption of CCD sensors is higher than the CMOS ones. In terms of image quality and sensitivity to light, CCD sensors have advantages over CMOS sensors. An extension of the 2D optical set-up consists in using two synchronized cameras (3D DIC) or multi-camera systems to reconstruct the entire surface deformation of an object [81].

For in situ measurements, in general, care must be taken in selecting the optical camera-lens system. Bratlett et al. [72] used a 3D DIC system consisted of standard two 5-megapixel CCD cameras coupled with 35 mm compact lenses with a sterovision angle of about 20${}^{\circ }$. The stereovision system was needed to measure full-field geometric distortions of printed parts resulting from residual stresses developed in the manufacturing process. In another study, Bratlett et al. [2] integrated a similar 3D DIC system into a DMLS machine during manufacturing. The cameras were mounted overhead through low-bandpass mirror viewports blocking saturated laser irradiation. Calibration of the stereo-DIC system was performed at the build level height before starting the build process, with a certain depth of focus. Lighting was provided by the machines internal overhead light-emitting diode (LED) system. A pair of images were taken for each powder layer during part production prior to laser melting of the layer. Holzmond et al. [71] used a 3D DIC system as a non-destructive in situ measurement technique to monitor the surface geometry of printed parts. The optical system consisted of standard 5 megapixel CCD cameras with 50 mm compact lenses. A simple desk lamp and a diffused LED lamp were employed for local illumination. For image quality enhancement, the LED lamp was positioned from above and the desk lamp at an approximate 42${}^{\circ }$. This configuration provided the required illumination when working with a small lens aperture. Biegler et al. [73] used the commercial 3D DIC GOM Aramis 4M coupled with optical filters to measure in situ distortions directly on a wall geometry produced with LMD. The stereovision system was set to a distance of 555 mm from the object with a base distance of 216 mm between the two cameras. Standard 50 mm lenses with an aperture of *f* 11 were used, resulting in a measuring volume of 150 × 110 × 64 mm${}^{3}$. The measuring volume was calibrated with a target plate adjusted to the area of interest. The lenses on both cameras were mounted with a narrow band-pass interference filter (wave-length of 810 nm ± 22.5 nm) to blockout the bright light occurring during the welding process. In order to have sufficient light for the optical measurements, an 808 nm defocused monochromatic laser beam from a DILAS compact diode laser was used. This DIC-based set-up was also used by the research group in other studies [74,75,82]. Xie et al. [76,78] successfully obtained full-field strain measurements on a manufactured thin-wall during the LENS AM process using 2D DIC. A CMOS HS-UX50 160 K camera coupled with a standard Nikon Nikkor 60 mm f/2.8D lens was used. A fixed light source was used to uniform illumination and an optical filter was installed in front of the lens. In these studies, commercial DIC software were used. Namely, the two most common software were Vic-3D (Correlated Solutions) [2,71,72], Vic-2D (Correlated Solutions) [76,78] and GOM Aramis 3D DIC [73,74,75,79]. These turn-key DIC systems have been used with minor adjustments in terms of lighting and filtering. Nevertheless, a more flexible optical system may be required to improve, for instance, the spatial resolution of the target area of interest across the field of view. A summary of the DIC optical and lighting systems employed with regard to the different AM variants is given in Table 2.

#### 3.2.2. Speckled Pattern

In the 2D/3D DIC technique, the deformation is assessed by correlating the geometrical deformation of a textured pattern which is assumed perfectly bonded to the material in a way that it can precisely describe its surface deformation. Typically, to perform the image correlation, a random speckled pattern is created and imaged over the region of interest with suitable quality in terms of granular size, contrast and isotropic feature-orientation. It is recognized that the quality of the speckled pattern has a significant influence on the accuracy and spatial resolution associated with DIC measurements [99]. A survey of different procedures to create the suitable patterns at different scales; experimental conditions and material surfaces can be found in [100,101,102].

The creation of a speckled pattern, however, is a challenge in most AM processes. For instance, in the AM processes, high temperatures are reached during the material deposition which can prematurely damage standard aerosol spray and airbrush painting procedures. This issue can be mitigated by using paintings materials that are stable at high temperatures [73,74,103]. In some studies, the stained natural patterns of the material surface is directly used for DIC processing [71]. However, this approach is only possible to apply for certain materials and in samples manufactured by powder bed fusion (PBF) processes. In other studies, materials with a high melting point were used to fabricate the stain pattern. However, there are processes where it is almost impossible to apply a suitable pattern such as the Vat photopolymerization process, where the part to be manufactured is immersed in a liquid. Dong and Pan [104] manage to create a natural texture by illuminating a rough sample surface using an ultraviolet light in studying ablative materials at high-temperature by in situ stereo-DIC.

In different AM processes, both natural and painted speckled patterns have been proposed to create the suitable textured image required in the DIC technique. Bartlett et al. [2,72] pointed out that the natural textured surface on the SLM pieces provided sufficient contrast to carry out the image correlation. However, such natural pattern may not be optimum with regard the DIC algorithm, but eventually overcomes the difficulty in painted the required pattern in this case. Xie et al. [76,78] have also taken advantage of the natural pattern of the manufactured sample, overcoming the difficulties in preparing the speckled pattern ahead of time across the part surface. The tough surfaces of the formed parts served as the natural pattern in the DIC procedure. It was only possible to take advantage of the natural pattern in the surface of the samples produced by PBF AM process because of its feedstock being powders. Nevertheless, in this simple approach, it is not guaranteed to obtain an optimum textured pattern, in terms of contrast and speckled features, for image correlation. In Biegler et al. [73,74], a high contrast stochastic pattern was painted to the sample to track transient deformations and distortions. The AM process was interrupted to coat the sample with the speckled pattern for DIC measurement purposes. Additional layers were then deposited continuing the AM process. The speckled pattern was applied using boron nitride as a white primer and black iron oxide powders (II, III) for the random granular pattern. Both paintings were stable at high temperatures and have good adhesion to the material substrate. He et al. [79] has created a speckled pattern, with high-temperature resistance and high-contrast, by painting a white primer followed by black iron oxide powder. Holzmond et al. [71] used a method that explores the natural pattern of the piece by adding ColorFabb Woodfill Fine filament in the AM process. The printed material was a mixture of recycled wood fiber of 10 to 20% by weight, poly lactic acid (PLA) and poly (hydroxyalkanoate). The average roughness of ColorFabbWoodfill was 1.39 μm, and the average roughness of PLA (ToyBuilder Labs) was 0.27 μm. The value of 0.27 μm for the PLA-wood parts seems very low. Indeed, in Alsoufi and Elsayed [105], it is reported that the average roughness surface ranges among 1.5 to 4.74 μm when measured in the direction of material deposition, and in between 9.44 and 32.78 μm when measured perpendicularly. In addition, the resulting speckled pattern, despite being sufficient to carry out the image correlation, may not be optimal with regard to contrast, signal-to-noise ratio and spreading over the dynamic of the digital sensor. The motivation was to avoid adding a layer of paint on each new printed layer, changing material properties of the final part and adding a step to the 3D printing process. Material extrusion is the only process in which it is possible to use a filament with fibres that allows the marking with sufficient contrast for DIC measurements. Table 2 complements the summary of these studies in terms of speckle pattern in DIC measurements as a function of AM variants.

#### 3.2.3. Radiation

One major concern when carrying out in situ DIC measurements in AM is the high radiation that typically takes places on the process. This is a challenge with regard to standard procedures at the macro scale in several engineering applications, where the simple lighting systems are eventually suitable with no need for special optical accessories. The issue of high radiation is more pronounced on the DED and PBF AM processes. In DED, the radiation comes from the deposition process of the feedstock. This radiation emanating from the heat source used in the process affects the molten pool and its proximity, making this area very bright. In PBF, laser radiation is emitted by the formation of the molten pool during the melting of the powder bed. This radiation travels directly towards the cameras during in situ measurements, making the image acquisition suitable for DIC processing very difficult (e.g., to avoid pixel saturation). To overcome this issue, some solutions have been proposed in the literature. A basic approach can be used similar to different applications in machine vision to avoid unevenly contrasted images or reflections which simply consists of using filters. Hence, procedures have been reported using an optical filter in front of the camera lens, which limits the passage of light through its narrow band [73,74,75,76,78,79]. On the other hand, other optical components such as a low band hot mirror display have been used to block a harmful radiation towards the camera-lens system [2].

#### 3.2.4. Projected Particles

During the AM process, some particles from the deposition zone can be projected. The AM process in which there is more particle projection is the DED. A practical solution to protect the DIC system is to use an optical glass. He et al. [79] used a quartz glass to shielded lens from the attack by projected particles. Ocelík et al. [106] used a simple solution by applying a piece of cardboard positioned in the setup in order to protect the chambers from radiation and particles projected during the process.

#### 3.2.5. Camera Position

In the PBF process and the Vat photopolymerization process, the parts to be manufactured are immersed in powder and liquid, respectively, which makes it difficult or even impossible to capture images using a camera-lens optical system.

#### 3.2.6. Curved Objects and Out-of-Plane Deformations

The simplest optical system for in situ measurements with DIC requires only one camera (monovision system). However, advanced monitoring on parts with curved geometry or out-of-plane deformations require the utilisation of a 3D DIC set-up employing two synchronised and calibrated cameras (stereovision system). The AM processes where this difficulty is verified are the DED and Material jetting processes.

#### 3.2.7. Closed Process Chamber

Some of the AM processes need a closed chamber for the procedure to take place. This closed chamber requires an inert atmosphere, using gases such as nitrogen or argon, which allows the processing of a wide variety of materials. This condition brings an extra difficulty when carrying out in situ DIC measurements. In Bartlett et al. [2], the cameras of a DIC 3D system were installed overhead of the machine for in-process monitoring. A low-band hot mirror window was placed between a closed chamber and the cameras to preserve the inert atmosphere and to protect the cameras from radiation emitted in the process. The AM processes where this difficulty occurs are PBF, Vat photopolymerization and binder jetting, as these require a closed chamber for the manufacture of parts, which makes it difficult to acquire images.

#### 3.2.8. Relative Motion

During DIC measurements, the optical system is typically stationary with regard to the process under analysis. However, in some AM processes, such as PBF, material jetting, binder jetting, material extrusion and sheet lamination, this condition may be difficult to guarantee during the process, and the optical system may need to be fixed to the torch or other apparatus.

## 4. In Situ Monitoring of WAAM Using DIC: Challenges and Solutions

In this section, a case study is presented on in situ monitoring of the WAAM process using DIC, presenting preliminary results to highlight challenges and solutions.

Among all AM process variants, WAAM is one of the most promising [88,107]. WAAM allows a high deposition rate to produce large and complex parts for structural applications. Many different metallic alloys can be used, such as steel, titanium, aluminum, or magnesium. Additionally, WAAM can be implemented at a relatively low cost, using conventional MIG/MAG welding machines, together with an XYZ Cartesian positioning system or a robotic arm.

WAAM involves the deposition of successive layers of molten metal, creating many thermal cycling in the layers below. Consequently, high residual stress can be generated, producing distortions in the parts. Additionally, defects can occur, such as porosities, lack of fusion, or cracks. Therefore, the in situ process monitoring is of primary importance, to detect and fix such issues in time. Several NDT has been studied for inline and offline inspection [108,109,110,111] but other reliable monitoring technologies must be developed or adapted for wider industrial implementation. DIC can be a valuable technology for in situ monitoring of WAAM, but several adverse process conditions prevent the use of DIC during the production of parts by WAAM. Such adverse conditions include:

- High-intensity electromagnetic radiation: the open electric arc produces a plasma (5000 to 30,000 ${}^{\circ }$C) that radiates in the infrared, visible and ultraviolet wavelengths. This invalidates the use of basic image acquisition optical set-ups for DIC measurements;
- High temperature reached in the inspection surface: the melting pool (>1000 ${}^{\circ }$C) produced during WAAM heats the metal surface preventing the use of conventional painted speckled patterns;
- Sparks and projection of melted metal: near the material deposition zone, an intense projection of incandescent metal particles and fume may exist. This prevents positioning the camera near the target surface and also interferes with image acquisition;
- Relative movement between camera and target inspection zone: during the material deposition, the WAAM torch moves along the part being produced, making a continuous shift of the target inspection surface.

These issues are further discussed and illustrated, resulting from an experimental application of DIC during the production of a WAAM steel sample. A general overview and recommendations are presented. The sample was produced using a Metal Active Gas (MAG) welding power source PRO MIG 3200 from KEMPY. The wire feed speed was about 4 m/min, the travel speed was about 350 mm/min and the length of the produced samples was 130 mm, using a 1 mm wire diameter AISI316L stainless steel as a feedstock material. The voltage and the electric current prescribed were 20 V DC and 120 A, respectively.

**Figure 4:**
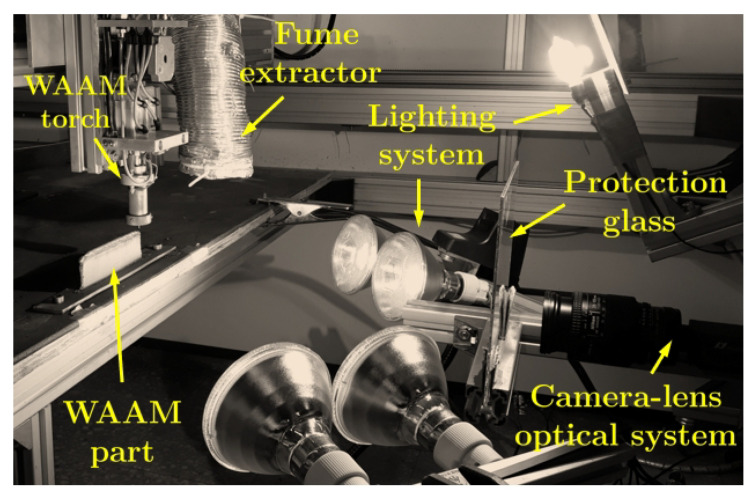
WAAM and DIC set-ups.

The speckled pattern was painted across the region of interest of the part that was firstly manufactured by WAAM, serving as the base for further layer deposition in a two stage procedure approach, similar to the one reported in [74]. The optical system consisted of a Manta G-1236 Allied Vision CMOS camera wit a Nikon AF Nikkor 28–105 mm f/3.5–4.5 D (IF MACRO) lens. The optical set-up coupled with the WAAM apparatus is shown in Figure 4. The DIC analysis was carried out using the MatchID software [57]. In this study, the following DIC setting parameters were used: subset size of 41 × 41 pixels; subset step of 10 × 10 pixels; ZNSSD correlation criterion; bicubic splines image grey level interpolation; affine shape functions; strain window of 5 × 5 control points; displacement approximation using bilinear (Q4) Lagrange polynomials; Green–Lagrange strain calculation algorithm.

**Figure 5:**
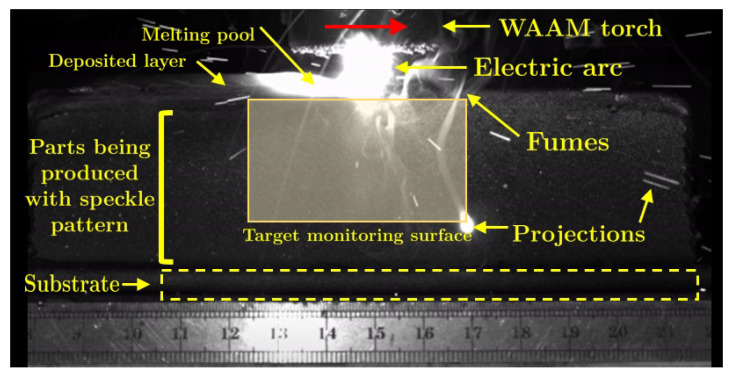
Acquired original image of the WAAM process for DIC purposes.

**Figure 6:**
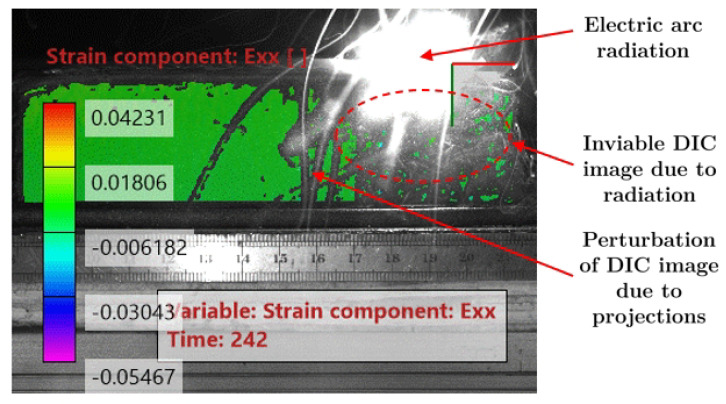
Strain in the horizontal direction calculated from the original image of Figure 4.

**Figure 7:**
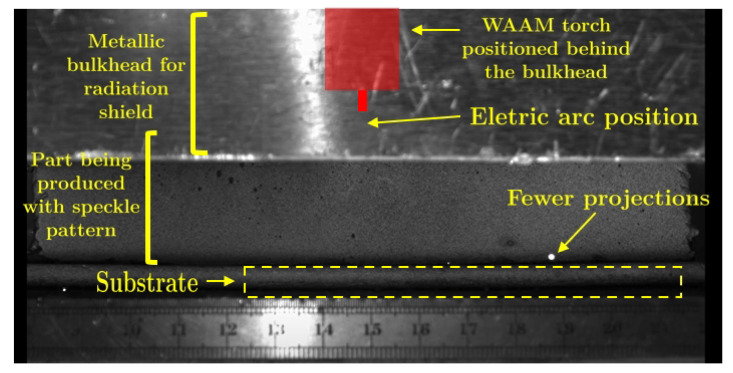
Acquired original image of the WAAM process for DIC purposes. In this case, a metallic bulkhead was used for radiation shield and to reduce the projections.

**Figure 8:**
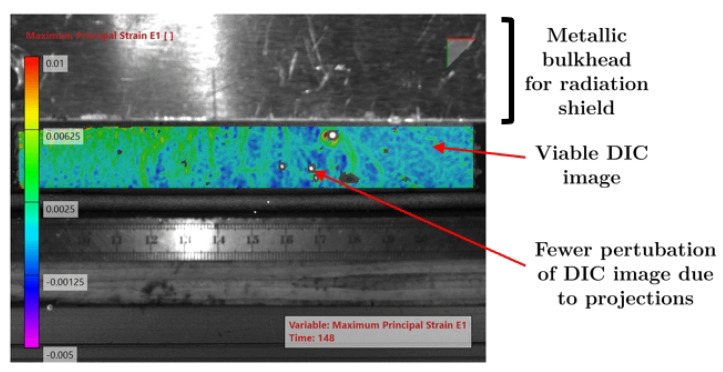
Principal strain calculated from the original image of Figure 6.

Figure 5 presents the original image of the WAAM acquired for DIC purposes. The effect of the high-intensity radiation near the electric arc can be seen, disturbing the image. Consequently, the correspondent DIC image (Figure 6) is inviable. To avoid this problem, a metallic bulkhead was placed in front of the electric arc to block the radiation and to reduce the projections (Figure 7). This procedure clearly improves the DIC image, as can be seen in Figure 8.

## 5. Conclusions

In this paper, a systematic review on in situ monitoring of AM processes using DIC full-field measurements was presented. The following remarks can be drawn:

- DIC has been successfully applied for in situ measurements in a few manufacturing processes, namely DED and PBF. The access of full-field measurements has been enhancing the optimisation of AM processes along with the quality of the manufactured part;
- Research studies using in situ measurements for monitoring AM processes can be grouped in one of the following topics: defect characterisation, evaluation of residual stresses, geometric distortions, numerical modelling validation;
- The 3D DIC set-up is typically selected to carry out in situ measurements on AM processes. This variant uses two calibrated cameras to register images during the process, which has advantages over the 2D-DIC system, since material deformation can occur generically in the 3D space (e.g., by the occurrence of geometrical distortions generated by residual stresses);
- LEDs and lasers are the two types of lighting systems used in in situ monitoring. Optical filter in the lenses of the optical system were also used to enhance image quality from the light radiation typically generated in AM processes;
- The creation of the textured pattern required in in situ DIC measurements was a major issue. On the one hand, a speckled pattern was created by painting using material that is stable at high temperatures. On the other hand, the use of natural textured patterns imaged directly over the surface of the material was explored, with the advantages of not adding an interruptive step to the process. In some AM processes, eventually, an additional material can be added in the procedure to enhance the natural surface. These patterns, however, may not be fully optimised regarding the accuracy associated with the DIC measurements;
- When applying in situ DIC measurements in AM processes, the following challenges were identified and discussed: optical and lighting system, speckled pattern, radiation, projected particles, camera position, curved objects and out-of-plane deformations, closed process chamber, relative motion;
- A case study on the WAAM process using a 2D DIC MatchID system was presented. Preliminary results were reported showing the feasibility of in situ DIC measurement in this process. The effect of the high-intensity radiation near the electric arc was discussed and a solution presented using a metallic bulkhead placed in front of the electric arc to block the radiation and to reduce projections.

## Figures and Tables

**Table 1:** Summary of main difficulties in applying DIC to the different AM variants.

AM Technology	AM Variant	Radiation	Projected Particles	Camera Position	Speckled Pattern	Curved Objects	Closed Chamber	Relative Motion	Related References
	DMD	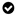	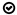	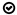	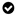	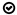	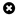	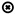	[83]
	3D Laser Cladding	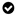	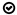	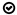	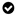	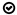	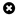	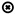	[84]
DED	DLD/LMD/LENS	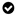	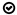	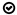	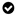	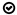	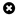	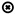	[85,86,87]
	WAAM	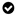	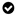	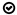	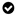	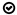	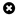	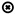	[88,89]
	SLS	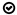	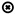	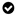	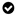	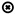	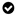	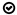	[90]
PBF	SLM/DMLS	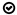	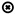	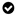	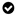	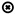	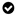	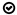	[90,91,92]
Vat photopolymerization		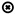	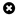	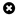	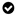	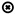	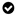	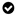	[93,94]
Material jetting		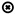	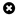	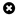	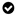	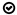	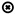	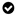	[93,95]
Binder jetting		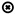	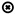	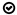	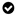	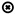	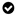	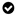	[93,96]
Material extrusion		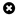	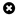	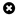	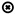	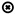	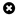	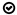	[93,97]
Sheet lamination		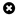	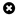	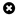	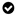	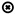	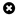	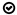	[93,98]

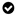
 Difficulty is verified. 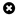 Difficulty is not verified. 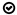 Difficulty is verified with exceptions. 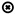 Difficulty is not verified with exceptions.

**Table 2:** Optical system, lighting and speckled pattern employed during AM variants.

AM Variant	Optical System	Lighting System	DIC Software	Speckled Pattern	References
SLM	Two 5-Mpx CCD cameras 35 mm compact lenses (sterovision angle of 20${}^{\circ }$)	LED	Vic-3D	Natural pattern	[2,72]
LMD	Two CMOS cameras	DILAS Compact diode laser	GOM Aramis 3D DIC	Painted stochastic pattern	[73,74,75]
DMD	Two CCD cameras	LED	GOM Aramis 3D DIC	Painted pattern	[79]
FFF/FDM	Two 5-Mpx CCD cameras 50 mm compact lenses	LED	Vic-3D	Natural pattern (using ColorFabb Woodfill Fine filament)	[71]
LENS	CMOS HS-UX50 160K camera coupled with a standard Nikon Nikkor 60	Fixed light source	Vic-2D	Natural pattern	[76,78]

## Data Availability

Not applicable.

## Abbreviations

The following abbreviations are used in this manuscript:

AM	Additive Manufacturing
CAD	Computer-Aided Design
CCD	Charge-Coupled Device
CMOS	Complementary Metal Oxide Semiconductor
DED	Direct Energy Deposition
DIC	Digital Image Correlation
DLD	Direct Laser Deposition
DMD	Direct Metal Deposition
DMLS	Direct Metal Laser Sintering
DVC	Digital Volume Correlation
ESPI	Electronic Speckle Pattern Interferometry
FDM	Fused Deposition Modeling
FFF	Fused Filament Fabrication
FFOTs	Full-Field Optical Techniques
FGM	Functionally Graded Materials
GM	Grid Method
LED	Light-Emitting Diode
LENS	Laser Engineered Net Shaping
LMD	Laser Metal Deposition
LMP	Laser Manufacturing Process
MI	Moiré Interferometry
MSL	Metal Shaping Laser
NDT	Non-Destructive Testing
PBF	Powder Bed Fusion
RS	Residual Stresses
SLM	Selective Laser Melting
SLS	Selective Laser Sintering
WAAM	Wire and Arc Additive Manufacturing
ZNSSD	Zero-Normalized Sum of Squared Differences

## Appendix A. Additive Manufacturing Processes

In AM, several distinct processes have been developed and are currently available on the market. Based on the fundamental parts and functionality of the equipment, these processes can be grouped into seven distinct categories [93]: (i) directed energy deposition; (ii) powder bed fusion; (iii) vat photopolymerization; (iv) material jetting; (v) binder jetting; (vi) material extrusion; (vii) sheet lamination. In the following, the principal characteristics of each process are described and reviewed.

### Appendix A.1. Directed Energy Deposition

Directed energy deposition (DED) technologies require a concentrated energy source and a flow of raw materials, crossing over a common focal zone. Typically, these processes have the presence of an inert shielding gas. The density of energy generated at a specific point melts the raw material in and around that place, giving rise to the formation of melt pools [112]. This procedure can be seen in the video [89].

#### Appendix A.1.1. Direct Metal Deposition

The technology of direct metal deposition (DMD) can also be termed by the laser manufacturing process (LMP) or metal shaping laser (MSL). This technology is aided by a laser and allows the manufacturing of materials by layered deposition, through a melting sequence, with powder solidification through the laser beam. This process also allows for other applications such as surface treatment (coating) or repair of parts [9,83].

#### Appendix A.1.2. 3D Laser Cladding

Laser cladding is a powder injection technology using a laser source. This technology allows the manufacture, repair of pears, coating and rapid metal prototyping. In this process, a laser beam melts the powder particles, forming a thin layer of substrate. A wide variety of deposition materials can be used [84].

#### Appendix A.1.3. Direct Laser Deposition

Direct laser deposition (DLD) can also be termed by Laser metal deposition (LMD) or Laser engineered net shaping (LENS) is based on a laser for the manufacture of functional metal parts. This process is carried out by simultaneously supplying metallic powder and laser energy focused on an area. A relatively high power laser is used to create a melt pool on the substrate, using an inert atmosphere, the powder being injected simultaneously [86]. One of the biggest problems of this process is the residual stresses due to the heating and cooling of the part during its manufacture [87].

#### Appendix A.1.4. Wire and Arc Additive Manufacturing

The WAAM process is defined as a combination of an electric arc used as a heat source and a wire applied as a raw material. The WAAM technique is currently being adopted by industry due to its advantages, higher deposition rates and manufacturing of larger parts in less time [88].

### Appendix A.2. Powder Bed Fusion

In powder bed fusion (PBF) technology, a source of energy, such as the laser or the electron beam, is used to digitize each layer of the powder already spread in order to selectively melt the material, thus forming the piece. This consists of an inert atmosphere or partial vacuum process in order to provide protection to the molten metal. In this technology, several parts can be built simultaneously so that the construction chamber can be fully used [90]. This procedure can be seen in the video [91].

#### Appendix A.2.1. Selective Laser Sintering

The selective laser sintering (SLS) process uses a poser source to sinter powder to produce pieces of powder materials using one or more lasers that selectively sinter surface particles, that is, layer by layer. This technology is carried out in a closed chamber [90].

#### Appendix A.2.2. Selective Laser Melting

Selective laser melting (SLM) also known as direct metal laser sintering (DMLS) is similar to the SLS process [113]. The complete melting of the powder bed particles occurs using one or more lasers [90,92].

### Appendix A.3. Vat Photopolymerization

This is defined by the liquid photopolymer in a vat that is cured selectively by light activated polymerization. This light is usually the UV radiation from lasers or lamps. The raw material for this process is in a liquid or pasty format [93]. This procedure can be seen in the video [94].

### Appendix A.4. Material Jetting

Material jetting is one of the fastest and most accurate AM technologies. This technology is defined as a process in which droplets of construction material are selectively deposited. These droplets can be a liquid photoplomer or a fused wax, and are solidified through a radiation light source by chemical reaction [114]. This procedure can be seen in the video [95].

### Appendix A.5. Binder Jetting

This is characterised by the selective deposition of a liquid bonding agent in order to join powder materials. Powders, powder mixtures and a liquid bonding agent are the raw materials for this technology. These raw materials are connected through a chemical bond or thermal reaction, thus forming the metallic, ceramic or polymeric part [93]. This procedure can be seen in the video [96].

### Appendix A.6. Material Extrusion

Material extrusion is the most popular, well-known and popular technology of all AM processes. The material used in the process is selectively distributed through a nozzle or orifice until the piece is obtained. The material used is a filament or paste, which is usually thermoplastic or structural ceramics [115]. This procedure can be seen in the video [97].

### Appendix A.7. Sheet Lamination

Sheet lamination uses sheets of material, which are glued together to form an object. These sheets can be made of different materials, such as metal, polymers or powdered ceramic material held together by a binder. The sheets of material are bonded through a thermal reaction or a chemical reaction bond, using ultrasound [116]. This procedure can be seen in the video [98].
